# Supplementary material for: The membrane-proximal external region of human immunodeficiency virus (HIV-1) envelope glycoprotein trimers in A18-lipid nanodiscs
Source: Commun Biol. 2025 Mar 15;8:442. doi: 10.1038/s42003-025-07852-z (PMC11910548; doi:10.1038/s42003-025-07852-z)
Supplement: Supplementary file 1 — Supplementary Information [file 42003_2025_7852_MOESM1_ESM.pdf]

Supplementary Information

The membrane-proximal external region of HIV-1 envelope glycoprotein trimers in amphipol-lipid nanodiscs

Yi Qi, Shijian Zhang, Kunyu Wang, Haitao Ding, Zhiqing Zhang, Saumya Anang, Hanh T. Nguyen, John C. Kappes, Joseph Sodroski & Youdong Mao

Supplementary Tables 1-3 Pages S2 – S4

Supplementary Figures 1 through 12 Pages S5 to S25

Supplementary References 1 through 19 Pages S26 to S27

Supplementary Table 1. Structural models of the trimeric HIV-1 Env MPER without anti-MPER antibodies bound.

PDB/ EMDB	HIV-1 Env	Method	Description	Reference
PDB ID: 6E8W	MPER-TM peptide in a nanodisc	NMR	Stalk–bubble conformation with a 16-Å C_α-C_α distance between residue 665 on adjacent protomers.	1
PDB ID: 6DLN	MPER-TM peptide in a phospholipid bilayer	NMR	Tripod conformation with a 53-Å C_α-C_α distance between residue 665 on adjacent protomers.	2
PDB ID: 7SC5 EMD-25022	Cytoplasmic tail-deleted SOSIP HIV-1_{BG505} Env (residues 1-723, T332N, poly-Arg cleavage site changes). The Env was expressed in baculovirus-infected 293F cells, purified with the PG9 bNAb and reconstituted into MSP1D1dH5 nanodiscs with POPC, POPG and brain polar extract.	Cryo-EM	The structure of the cleaved Env ectodomain (residues 31-662) was solved at 3.9-Å resolution. Residues 663-671 from the NMR structure of an MPER peptide in DPC micelles (PDB ID: 2PV6) were fitted into the EM density map, providing an ~ 5-Å model of MPER residues 663-671.	3
EMD-21413	Full-length HIV-1_{BaL} Env trimers on aldrithiol (AT-2)-inactivated viral particles, complexed with 10-1074 and 3BNC117 Fabs	Cryo-ET	Fab-bound structures were solved at 9.9-Å resolution. The MPER forms a thin central stalk that connects the ectodomain and the membrane.	4
PDB ID: 7SKA EMD-25178	HIV-$1_{ADA.CM.v4}$ stabilized Env trimers on AT-2- inactivated viral particles	Cryo-ET	C3-symmetrized Env structure solved at 9.1-Å resolution. The gp41 α9 helix is short and ends at Gln 653; the remaining α9 residues (654-664) bend and form a thin, flexible 10-Å stalk connecting the ectodomain to the membrane. The entire MPER (residues 665-683) appears to be buried in the membrane	5

Supplementary Table 2. Comparison of Cα-Cα interprotomer distances (Å) in trimeric MPER structures.

Cα-Cα distance	Hypothetical composite model			6DLN (NMR) ** tripod-like[2]	6E8W (NMR) ** stalk-bubble[1]
	P1 & P3	P1 & P2	P2 & P3		
Near the MPER N-terminus (Lys 665)	29.3	28.4	29.0	54.4(0.9)<br>52.4(0.8)<br>47.3(0.6)	15.7(0.6)<br>15.8(0.6)<br>15.8(0.5)
At the entrance of the gp41 MPER into the membrane (Lys 683 → Ile 684)	*	26.0 → 25.2	*	17.3(0.3)→17.6(0.3)<br>17.1(0.5)→ 17.9(0.5)<br>18.2(0.3)→18.6(0.2)	9.7(0.6) → 15.4(0.4)<br>9.7(1.0) → 15.2(0.6)<br>9.8(0.8)→ 15.2(0.5)
In the membrane (Phe 685)	*	22.8	*	12.8(0.4)<br>13.0(0.3)<br>13.4(0.2)	13.7(0.7)<br>13.5(0.5)<br>13.7(0.6)
At the C-terminus of the HR1C coiled coil (Trp 596)	19.8	20.0	20.8		

* These chain F (Protomer 3) residues were not modeled in the composite Tri FPPR Env structure.

** The average distances, with standard deviations in parentheses, are provided for the NMR structures.

Supplementary Table 3. Overlap between Tri FPPR Class A/Class B and the original class3/class 6 populations.

	Class A (80,206 particles)	Class B (158,232 particles)
Original class 3 (99,585 particles)	0.33	0.43
Original class 6 (146,922 particles)	0.63	0.55
Total percentage	0.96	0.98

a

	Env (-)	wt AD8	AE2	Tri FPPR
HIV-1 strain of origin	JR-FL	AD8	AD8 (modified)[6]	AD8 (modified)[7]
Env-producing cells	CHO	A549	A549	A549
Env cleavage	No	Yes (76%)	Yes (91%)	Yes (96%)
Ligand	BMS-806 (added during Env synthesis)	BMS-806 (added to membrane prep)	BMS-806 (added to membrane prep)	BMS-806 (added to membrane prep)
Crosslinker	BS3	None	DTSSP	None
Solubilization	Cymal-5, A8-35	SMA	SMA	Amphipol A18
Counterselection	None	19b (V3)	19b (V3) +F240 (gp41)	19b (V3) +F240 (gp41)

b

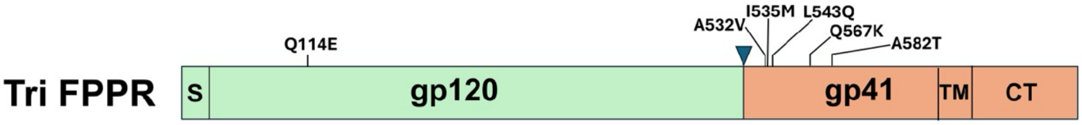

c

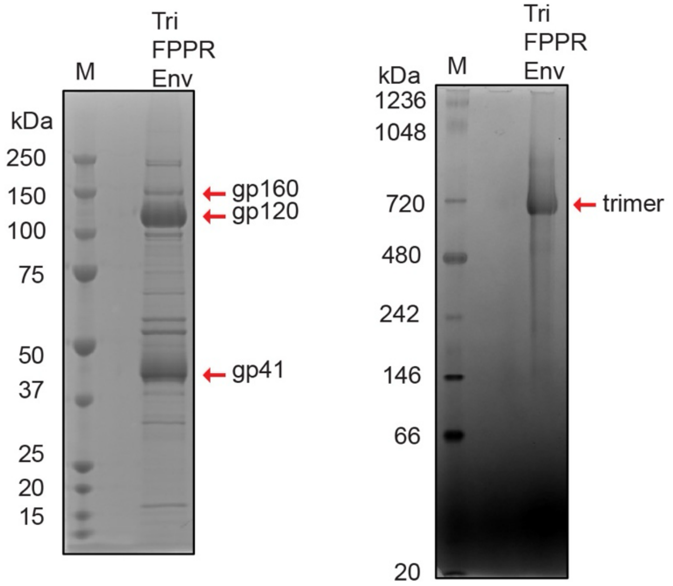

Supplementary Fig. 1. Preparation of the Tri FPPR Env trimers. a. Variables related to the preparation of the HIV-1_{AD8} Tri FPPR Env trimers are compared with those used for the preparation of the uncleaved Env(-) and cleaved wild-type (wt) HIV-1_{AD8} and AE2 Envs. **b.** A schematic diagram of the Tri FPPR Env is shown, with the cleavage site between the gp120 exterior Env subunit and the gp41 transmembrane Env subunit indicated by a triangle. The changes introduced into the HIV-1_{AD8} Env to stabilize the PTC[7] are shown above the diagram. Three of the Env changes (Q114E, Q567K and A582T) are designated "Tri"[6,7] and three of the changes (A532V, I535M and L543Q) in the fusion peptide-proximal region are designated "FPPR"[7]. S - signal peptide, TM – transmembrane region, CT – cytoplasmic tail. **c.** The Tri FPPR Env was purified from the membranes of A549 cells solubilized in Amphipol A18. The purified Tri FPPR Env was analyzed on a reducing SDS-polyacrylamide gel (left) and on a Blue Native polyacrylamide gel (right). M – molecular weight markers.

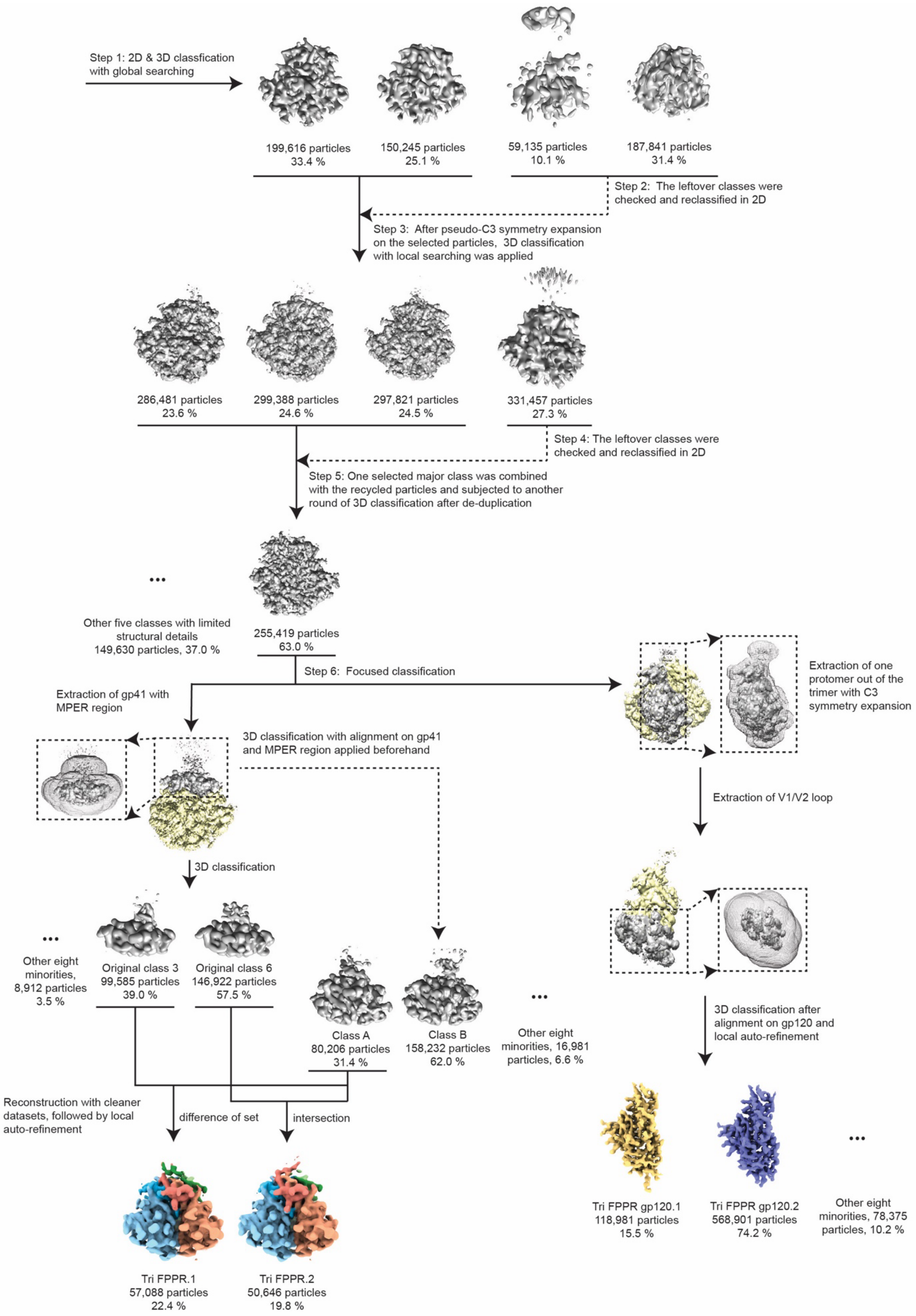
Step 1: 2D & 3D classfication with global searching
199,616 particles 33.4 %
150,245 particles 25.1 %
59,135 particles 10.1 %
187,841 particles 31.4 %
Step 2: The leftover classes were checked and reclassified in 2D
Step 3: After pseudo-C3 symmetry expansion on the selected particles, 3D classification with local searching was applied
286,481 particles 23.6 %
299,388 particles 24.6 %
297,821 particles 24.5 %
331,457 particles 27.3 %
Step 4: The leftover classes were checked and reclassified in 2D
Step 5: One selected major class was combined with the recycled particles and subjected to another round of 3D classification after de-duplication
...
Other five classes with limited structural details 149,630 particles, 37.0 %
255,419 particles 63.0 %
Step 6: Focused classification
Extraction of gp41 with MPER region
3D classification with alignment on gp41 and MPER region applied beforehand
Extraction of one protomer out of the trimer with C3 symmetry expansion
Extraction of V1/V2 loop
3D classification
Other eight minorities, 8,912 particles 3.5 %
Original class 3 99,585 particles 39.0 %
Original class 6 146,922 particles 57.5 %
Class A 80,206 particles 31.4 %
Class B 158,232 particles 62.0 %
Other eight minorities, 16,981 particles, 6.6 %
3D classification after alignment on gp120 and local auto-refinement
Reconstruction with cleaner datasets, followed by local auto-refinement
difference of set
intersection
Tri FPPR.1 57,088 particles 22.4 %
Tri FPPR.2 50,646 particles 19.8 %
Tri FPPR gp120.1 118,981 particles 15.5 %
Tri FPPR gp120.2 568,901 particles 74.2 %
Other eight minorities, 78,375 particles, 10.2 %

Supplementary Fig. 2. Cryo-EM data classification workflow. The diagram illustrates the major 3D classification steps for the Tri FPPR dataset. Focused 3D classification[8] of the gp41 portion produced the Tri FPPR.1 and Tri FPPR.2 maps. Focused 3D classification of the gp120 portion produced the gp120.1 and gp120.2 maps.

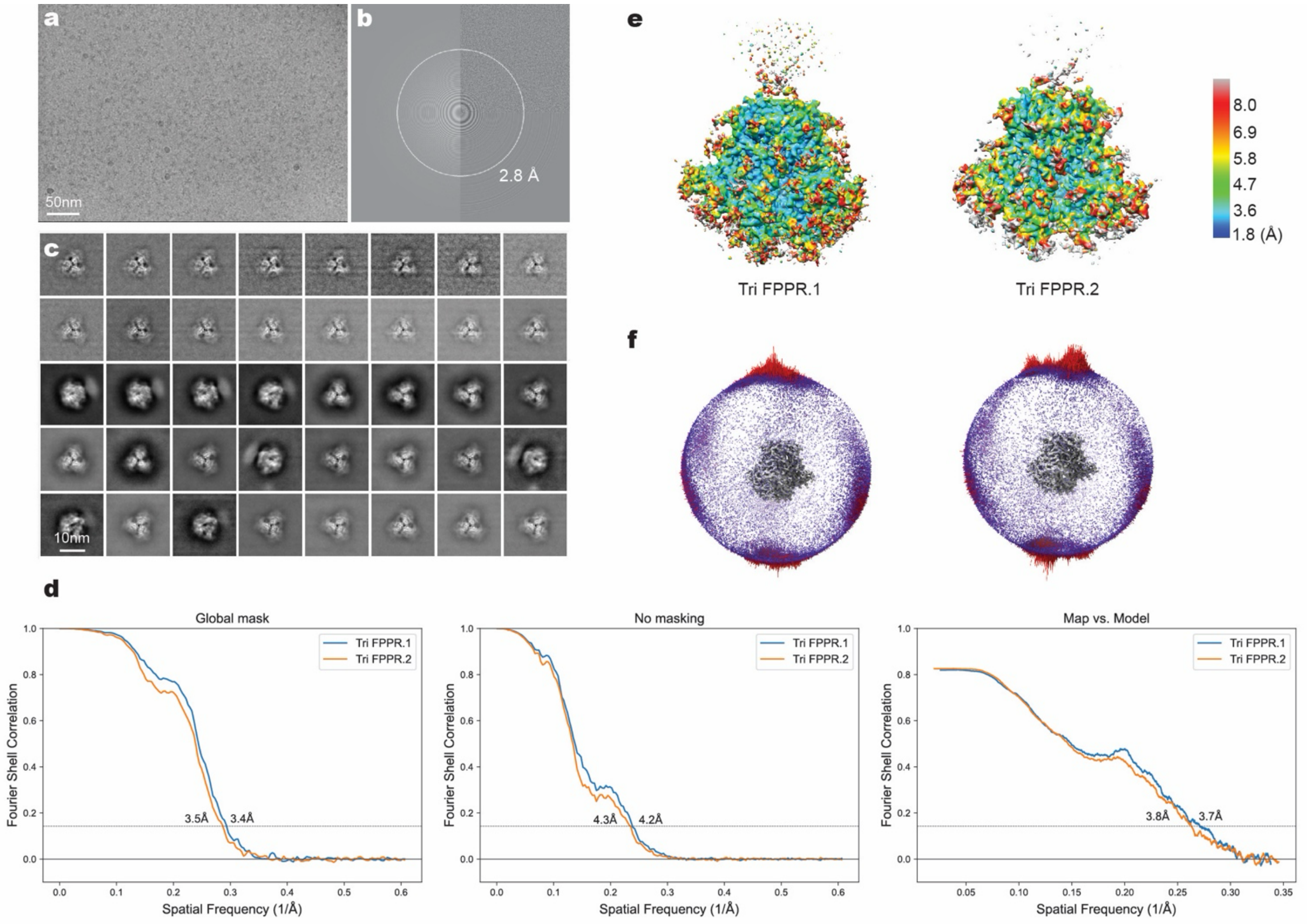

Supplementary Fig. 3. Cryo-EM analysis of the Tri FPPR.1 and Tri FPPR.2 Envs. a. A typical cryo-EM micrograph of the Tri FPPR Env trimers is shown. **b.** A Fourier transform of the micrograph in **a** was generated in Gctf[9]. **c.** Selected averaged images of the Tri FPPR Env trimers from unsupervised 2D classification are shown. **d.** Gold-standard Fourier Shell Correlation (FSC) plots of the Tri FPPR.1 and Tri FPPR.2 cryo-EM maps are shown. The resolution was estimated at an FSC cutoff of 0.143. **e.** The local resolution of the Tri FPPR.1 and Tri FPPR.2 maps was measured by Resmap[10]. The maps are colored according to the local resolution, indicated by the color gradient key. **f.** The orientation distribution of the particles used for the reconstruction of the Tri FPPR.1 and Tri FPPR.2 maps is shown.

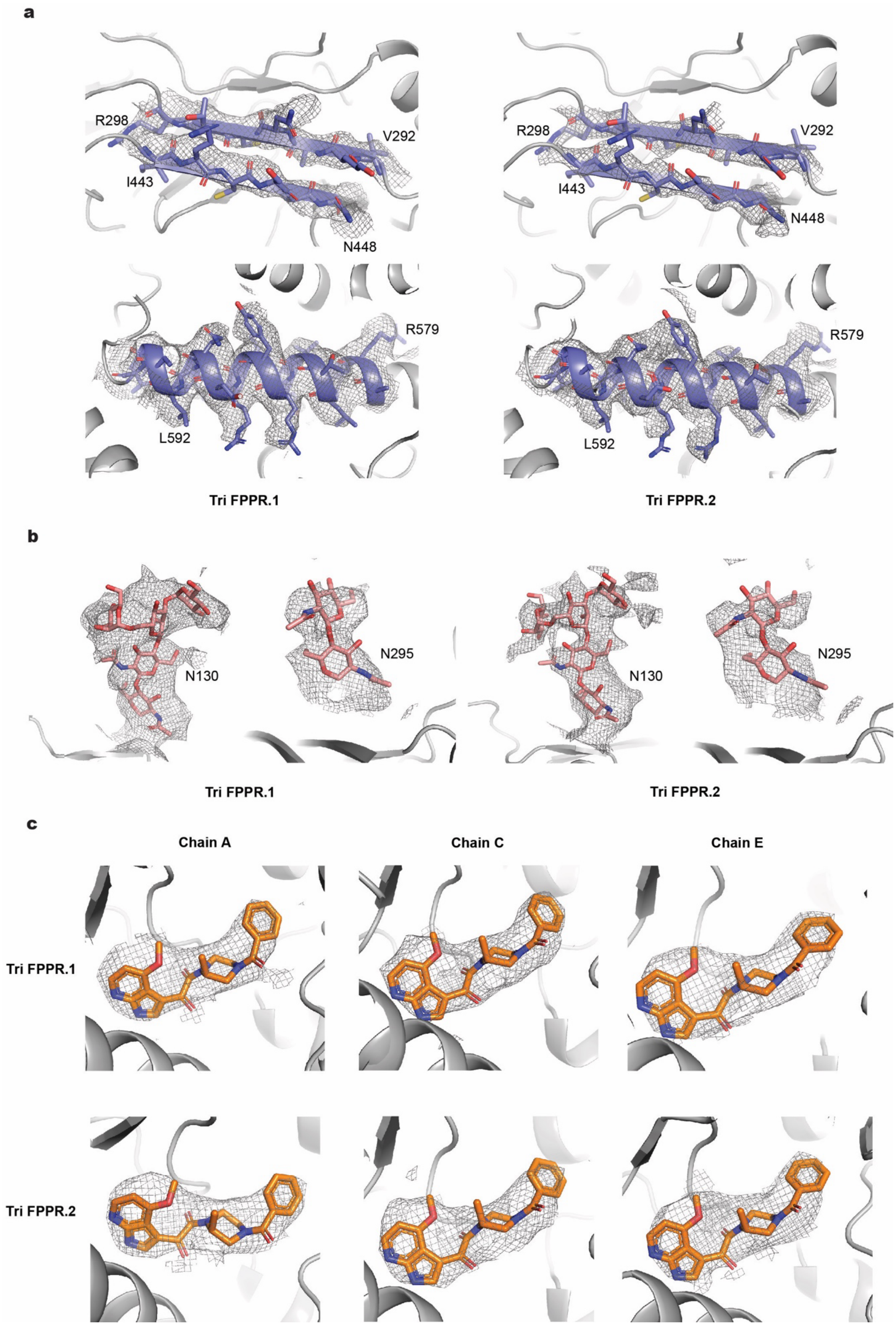
a
R298
V292
I443
N448
R579
L592
Tri FPPR.1
R298
V292
I443
N448
R579
L592
Tri FPPR.2
b
N130
N295
Tri FPPR.1
N130
N295
Tri FPPR.2
c
Chain A
Chain C
Chain E
Tri FPPR.1
Tri FPPR.2

Supplementary Fig. 4. Quality of the Tri FPPR.1 and Tri FPPR.2 maps. a. Representative features in the Tri FPPR.1 and Tri FPPR.2 models are shown along with the corresponding cryo-EM map density. The upper panels show gp120 β-strands and the lower panels shoe gp41 α-helices. **b.** Typical glycan models and associated map densities from Tri FPPR.1 and Tri FPPR.2 are shown. **c.** The modeled BMS-806 molecule and the associated map density is shown in all three gp120 subunits of the Tri FPPR.1 and Tri FPPR.2 structures. The position and orientation of the BMS-806 molecules are consistent with those in a previous cryo-EM structure of a solubilized membrane Env complexed with BMS-806[11] and in crystal structures of BMS-806 complexed with soluble gp140 SOSIP.664 Env trimers[12].

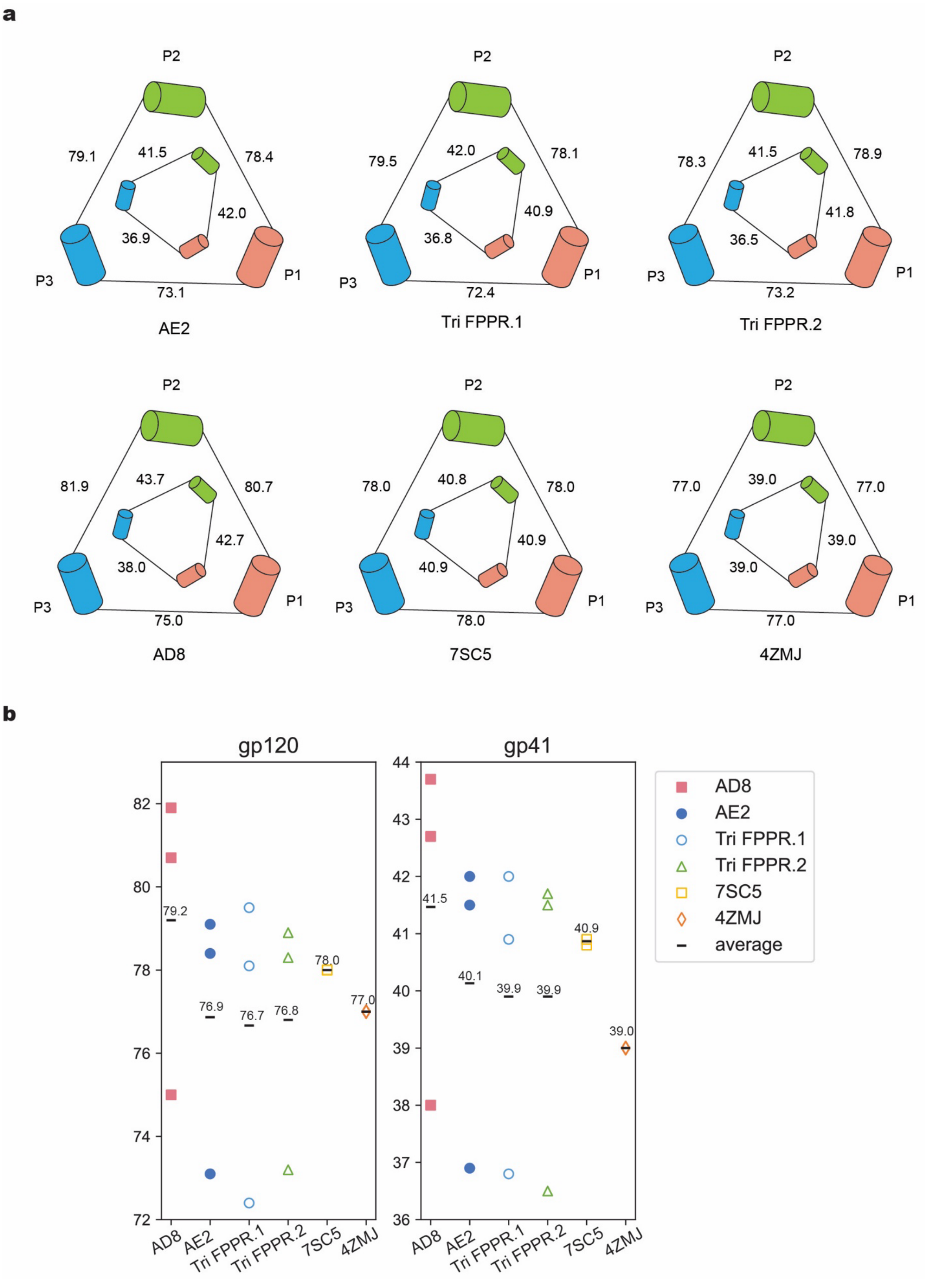
a
P2
P3
P1
79.1
78.4
41.5
42.0
36.9
73.1
AE2
79.5
78.1
42.0
40.9
36.8
72.4
Tri FPPR.1
78.3
78.9
41.5
41.8
36.5
73.2
Tri FPPR.2
81.9
80.7
43.7
42.7
38.0
75.0
AD8
78.0
78.0
40.8
40.9
40.9
78.0
7SC5
77.0
77.0
39.0
39.0
39.0
77.0
4ZMJ
b
gp120
gp41
AD8
AE2
Tri FPPR.1
Tri FPPR.2
7SC5
4ZMJ
average
79.2
76.9
76.7
76.8
78.0
77.0
41.5
40.1
39.9
39.9
40.9
39.0

Supplementary Fig. 5. Comparison of trimer geometry among different Env structures. a. The interprotomer distances (in Å) between selected Cα atoms (Thr 336 and Gln 352) of gp120 (outer triangles) and Cα atoms (Trp 628 and Ile 635) of gp41 (inner triangles) are shown for the wild-type HIV-1_{AD8} Env (PDB ID: 8FAD)[11], AE2 Env (PDB ID: 8FAE)[11], the Tri FPPR.1 and Tri FPPR.2 Envs, a membrane Env modified by SOSIP changes (PDB ID: 7SC5)[3] and an unliganded sgp140 SOSIP.664 Env trimer (PDB ID: 4ZMJ)[12]. **b.** The Cα-Cα distances shown in **a** are plotted for the gp120 and gp41 subunits. The black bars indicate the average distances.

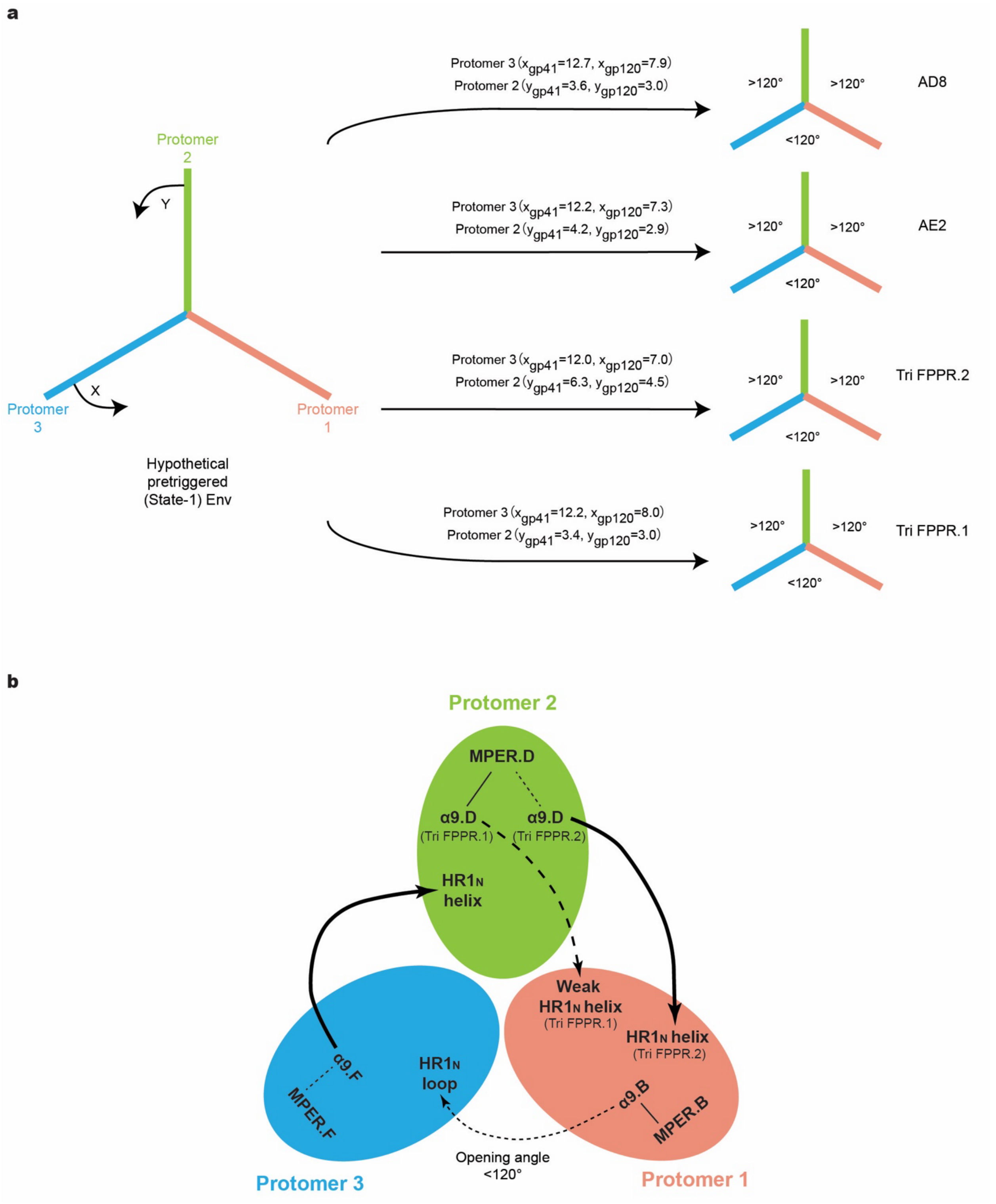

Supplementary Fig. 6. Asymmetry in the solubilized HIV-1 Env trimers. a. A model is shown for generating the observed asymmetric Env trimers from a hypothetical symmetric pretriggered (State-1) Env trimer. Beginning with a hypothetical C3-symmetric pretriggered (State-1) Env trimer, the asymmetric AD8, AE2, Tri FPPR.2 and Tri FPPR.1 Envs can be derived by the indicated rotational shifts in the gp41 and gp120 subunits of Protomers 2 and 3

(viewed from the perspective of the viral membrane). Note that the shifts in Protomer 3 (x values) exceed those in Protomer 2 (y values). Also, the shift in the gp41 subunit of Protomers 2 and 3 is greater than that of the gp120 subunit. **b.** A Tri FPPR Env trimer is schematically shown from the perspective of the viral membrane. The relationship of the MPERs, α9 helices and $HR1_N$ regions is shown. Based on the density in the cryo-EM maps, the connectivity of the gp41 MPERs to the adjacent α9 helices is depicted by lines joining these structures within each protomer. The strength of the connectivity ranges from weak (thin, broken lines) to strong (thick, solid lines). The stabilizing effect of the α9 helices on the $HR1_N$ helices in the clockwise protomers is depicted by curved interprotomer lines with arrows. Based on the α9 tilt angles and $HR1_N$ helicity, the stabilizing effects range from weak (thin, broken lines) to strong (thick, solid lines). Note that stronger association of the α9 helices with the adjacent MPER in the same protomer is inversely related to the stabilizing effect of the α9 helices on the $HR1_N$ helices in the clockwise protomers.

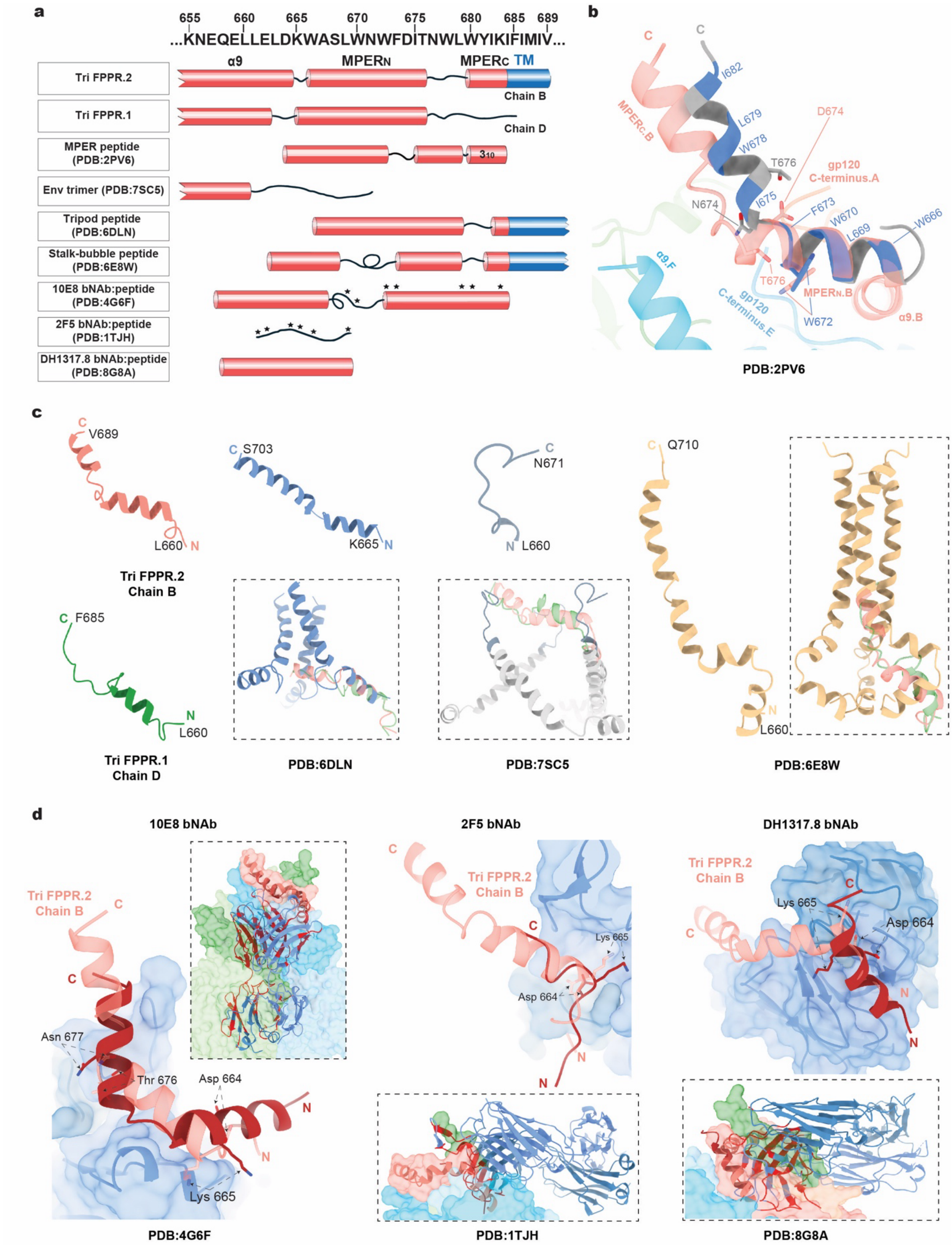

Supplementary Fig. 7. Comparison of the Tri FPPR MPER with other MPER structural models. a. The primary sequence of the Tri FPPR gp41 region near the membrane is shown.

Amino acids are numbered according to convention[13]. Secondary structures of Env proteins/peptides are shown. For the 2F5 and 10E8 bNAb:peptide complexes, the stars designate contact residues with the antibody[14,15]. **b.** A side view of the membrane-proximal base of the Tri FPPR.2 Env is shown, with Protomer 1 (chain B) colored salmon, Protomer 3 (chains E and F) cyan, and Protomer 2 green. Beginning with Asp 664, the MPERN helix of chain B Tri FPPR.2 Env was aligned with the NMR structure of an MPER peptide in DPC micelles (PDB ID: 2PV6)[16]. The residues of the 2PV6 MPER peptide (grey) that are buried in the DPC micelle are colored blue and define a hydrophobic surface that potentially interacts with the viral membrane. Side chains are shown for Trp 672, where the 2PV6 $MPER_N$ helix ends, and Thr 676, where the Tri FPPR.2 $MPER_N$.B helix ends. After $MPER_N$, the conformations and orientations of the hinge and C-terminal helices differ between 2PV6 and Tri FPPR.2. Note that in the Tri FPPR.2 Env, Asp/Asn 674, which regulates the stability of the pretriggered Env conformation[17], projects from $MPER_N$ towards the gp120 C-terminus, where it participates in a stabilizing salt bridge. In the aligned 2PV6 MPER, the corresponding Asn 674 projects in the opposite direction, away from any potentially stabilizing Env contacts. **c.** Tri FPPR.2 chain B (salmon) and Tri FPPR.1 chain D (green) MPER structures are compared to trimeric MPER structures without bound antibodies: a tripod NMR structure (PDB ID: 6DLN)[2], a solubilized SOSIP Env trimer reconstituted into nanodiscs (PDB ID: 7SC5)[3], and a stalk-bubble NMR structure (PDB ID: 6E8W)[1]. The individual protomers are shown in a similar orientation to those of the Tri FPPR MPERs. The trimeric MPERs are shown in the boxes; the Tri FPPR.2 chain B and Tri FPPR.1 chain D MPERs were aligned to the MPERs of 6DLN and 6E8W, and to the entire Env protomer of 7SC5. In no case was a satisfactory alignment possible. **d.** The structure of the Tri FPPR.2 chain B MPER (transparent, salmon-colored ribbon) was compared with crystal structures of MPER peptides (red) in complexes with bNAbs 10E8 (PDB ID: 4G6F)[15], 2F5 (PDB ID: 1TJH)[14] and DH1317.8 (PDB ID: 8G8A)[18] (bNAbs are shown as transparent blue surfaces). Selected residues are shown on both MPER structures. In the insets, the bNAb Fabs from the crystal structures are docked onto the composite Tri FPPR Env model, with the bNAbs oriented as seen in the bNAb-MPER peptide complexes. The bNAb Fab residues that clash with the composite Tri FPPR Env trimer (< 3.5-A Cα-Cα distances) are colored red, whereas bNAb Fab residues without clashes are colored blue. The surfaces of the composite Env trimers in the insets are colored as follows: red - Protomer 1; green - Protomer 2 and blue - Protomer 3. In all three examples, the bNAbs cannot bind the MPER of the composite Tri FPPR Env without significant clashes. This indicates that these bNAbs must recognize an MPER conformation that is different than that observed in the composite Tri FPPR Env trimer.

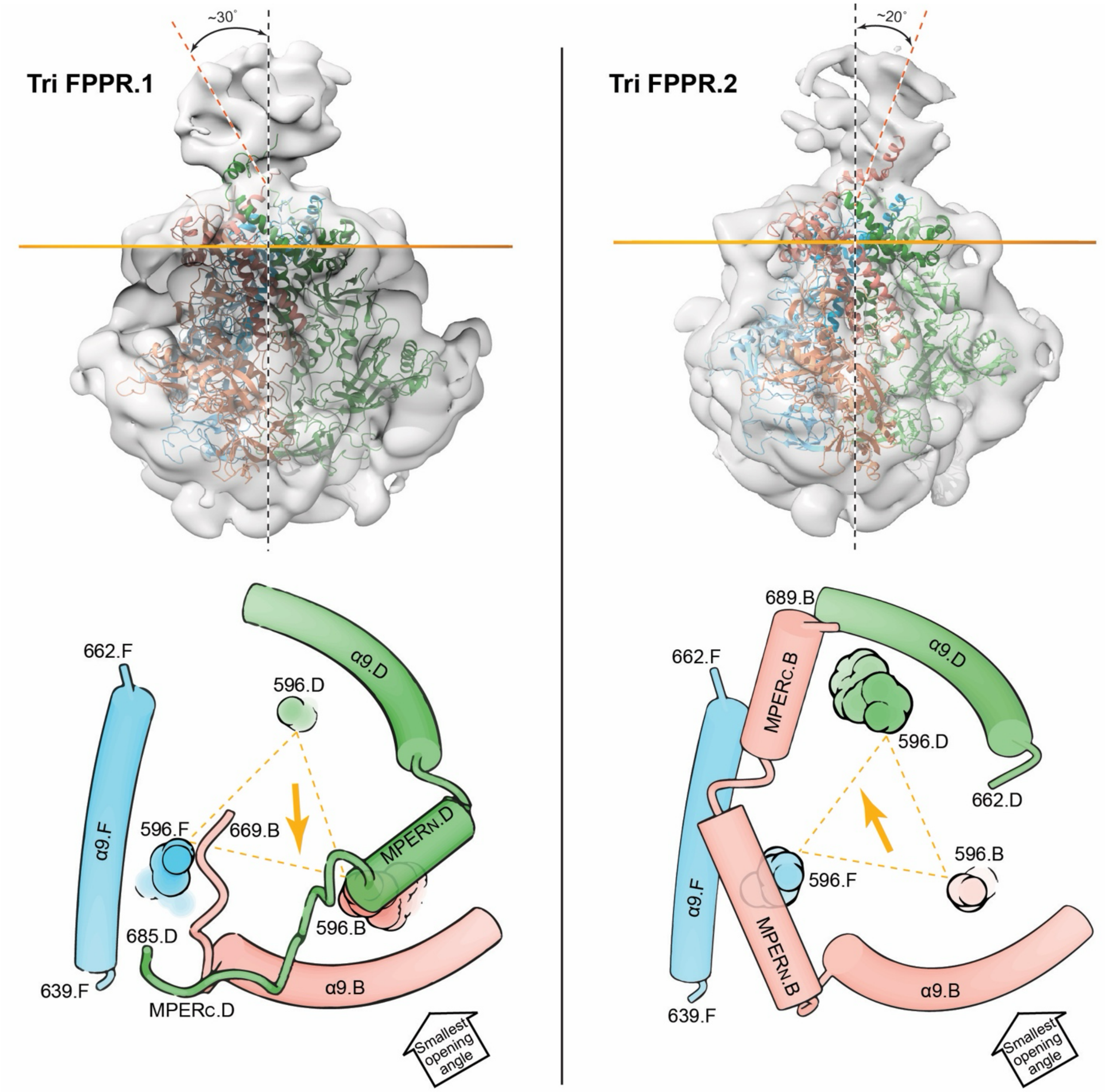

Supplementary Fig. 8. Relationship between Tri FPPR Env tilt in the A18-lipid nanodisc and MPER structure. In the upper figures, the Tri FPPR.1 and Tri FPPR.2 models are fitted into the 8-Å lowpass-filtered maps. The dashed red line is normal to the plane of the nanodisc. The yellow line indicates the position of a plane that passes through the three Trp 596 residues at the C-terminus of the $HR1_C$ coiled coil; the Env trimer pseudosymmetry axis (dashed black line) is normal to this plane. The tilt angles of the Tri FPPR.1 and Tri FPPR.2 Envs with respect to the nanodisc axis are 30 and 20 degrees, respectively. The lower figures show a view of the Tri FPPR.1 and Tri FPPR.2 Env models from the perspective of the nanodisc axis. For clarity, only the MPER and α9 elements and the Trp 596 residues are shown. The last resolved residues in the α9-MPER-TM regions of the Tri FPPR models are labeled. The Trp 596 atoms closer to the nanodisc plane are depicted as spheres of larger size. Also shown are the approximate directions of the Env tilt with respect to the nanodisc axis (yellow arrows). The

locations of the smallest opening angles between the gp120 subunits of the Tri FPPR.1 and Tri FPPR.2 Env trimers are indicated.

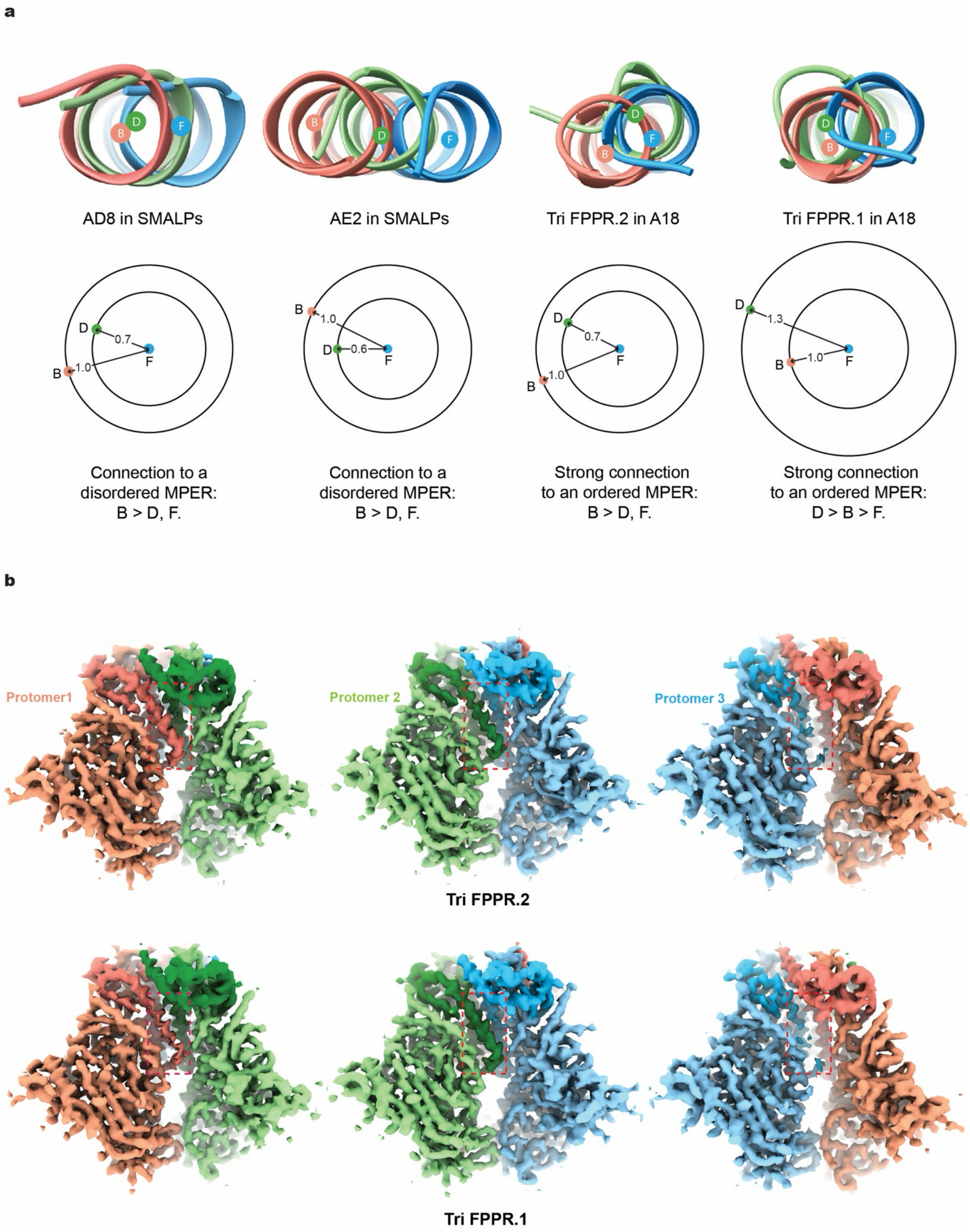

Supplementary Fig. 9. Relationship between the conformations of different elements of the asymmetric Env trimers. a. The tilt angles of the α9 helices in the different protomers of the asymmetric Env trimers are related to their connectivity to the MPER. The three protomers

of the indicated Env trimer models were aligned using the gp120 subunits. The C-terminal portions of the α9 helices of the three protomers are shown from the perspective of the viral membrane. The α9 helices are colored red (chain B, Protomer 1), green (chain D, Protomer 2) and blue (chain F, Protomer 3). The distances (in Å) from a centroid in the chain F helix to similarly positioned centroids in the chain B and chain D helices are shown in the lower images. The distances between the chain F and chain D α9 centroids were normalized to the chain F – chain B α9 distances in each model. The α9 helices further from the chain F helix exhibit stronger connections to more ordered MPER structures. **b.** Side views of high-contour maps of the Tri FPPR.1 and Tri FPPR.2 Envs are shown, with gp41 at the top and gp120 at the bottom of the image. Each of the three interprotomer interfaces is shown, with the $HR1_N$ region boxed. Chains A and B of Protomer 1 are colored red, Chains C and D of Protomer 2 are colored green, and Chains E and F of Protomer 3 are colored blue. The smaller opening angle (<120°) is between Protomer 3 and Protomer 1 of Tri FPPR.1 and Tri FPPR.2 (images on the right). Note that the $HR1_N$ region of Protomer 3 is a non-helical, poorly ordered loop that is not seen well at this contour level.

A comparison of the results in **a** and **b** indicates that in the Tri FPPR.1 Env, the α9 helices of chain B (Protomer 1) and chain D (Protomer 2) exhibit strong connections to the MPER, maintain tilt angles distinct from that of the α9 helix of Chain F, and are related to less helical $HR1_N$ regions on the clockwise adjacent protomers (Protomers 3 and 1, respectively) (viewed from the perspective of the membrane). In the Tri FPPR.2 Env, the α9 helices of chain D (Protomer 2) and chain F (Protomer 3) exhibit weak connections to the MPER, demonstrate similar tilt angles, and are related to strongly helical $HR1_N$ regions on the clockwise protomers (Protomers 1 and 2, respectively). Some aspects of these relationships are summarized in Supplementary Figure 6b.

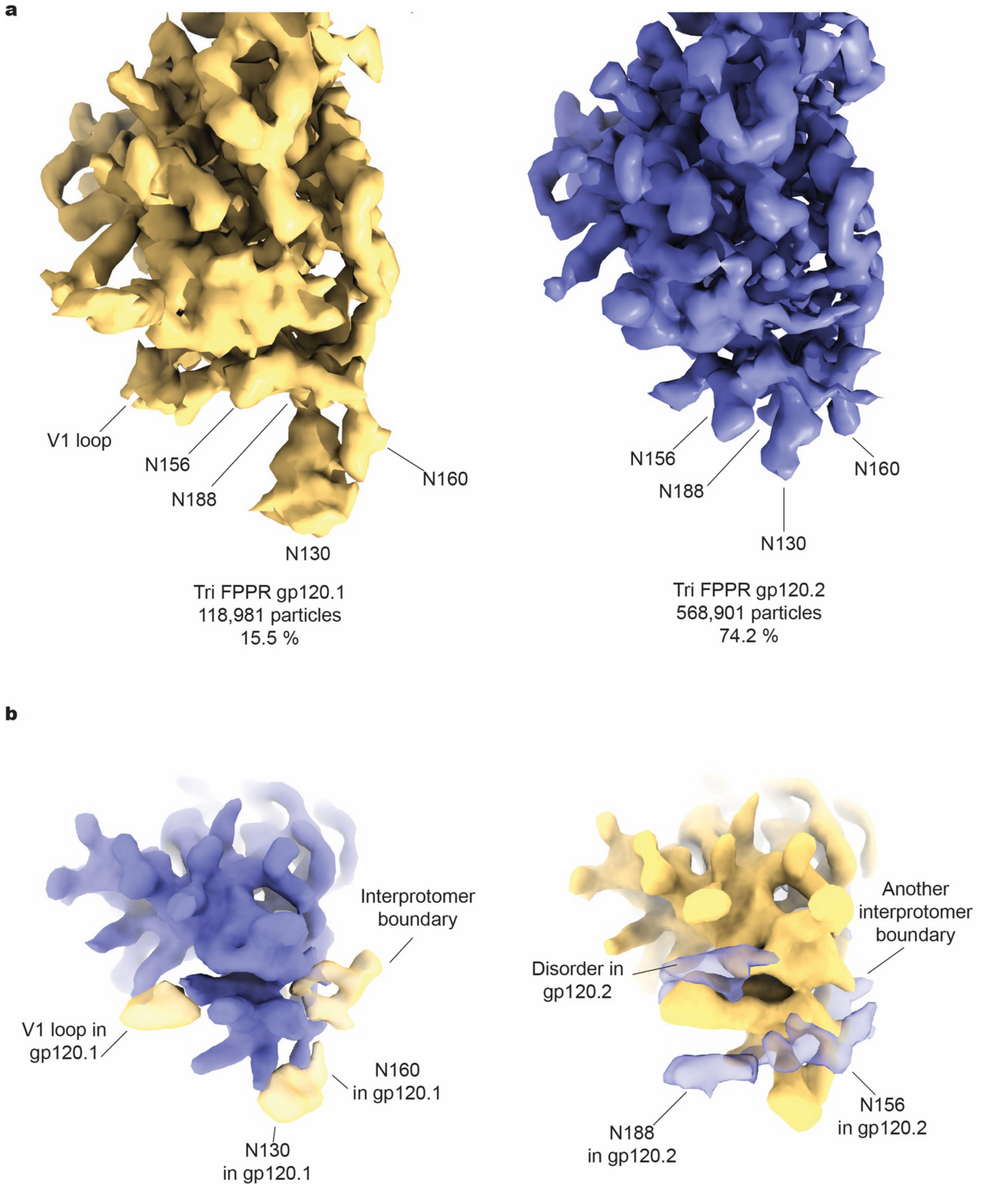

Supplementary Fig. 10. Glycan heterogeneity in the gp120 V1/V2 region. Focused classification[8] of the gp120 portion of the Tri FPPR Env maps yielded gp120.1 and gp120.2 maps that exhibit heterogeneity in the glycan-associated density in the gp120 V1/V2 region at the trimer apex. **a.** The Tri FPPR gp120.1 and gp120.2 maps are shown. The densities associated with glycans that differ between the gp120.1 and gp120.2 structures are labeled.

b. Difference maps highlight regions of density specific to gp120.1 (5σ differences in yellow in the left panel) and gp120.2 (4σ differences in blue in the right panel). Difference maps were generated with *vop scale* and *vop subtract* in Chimera[19]. As noted in the figures, some of the identified density results from the boundary with neighboring protomers or from disorder in the gp120.2 structure rather than from real differences between gp120.1 and gp120.2 Envs.

a

	[2,1,2]	[1,2,2]	[2,2,1]	[2,2,2]
**Data collection and processing**				
Magnification	105,000	105,000	105,000	105,000
Voltage (kV)	300	300	300	300
Electron exposure (e/Å)	51.7	51.7	51.7	51.7
Defocus range (μm)	–0.7 to –2.2	–0.7 to –2.2	–0.7 to –2.2	–0.7 to –2.2
Pixel size (Å)	0.825	0.825	0.825	0.825
Symmetry imposed	C1	C1	C1	C1
Initial particle images (no.)	1,540,890	1,540,890	1,540,890	1,540,890
Final particle images (no.)	26,633	14,023	25,921	105,509
Map resolution (Å)	4.2	4.6	4.2	3.4
FSC threshold	0.143	0.143	0.143	0.143
Map resolution range (Å)	2.5-10.0	2.8-10.0	2.6-10.0	1.8-8.0

b

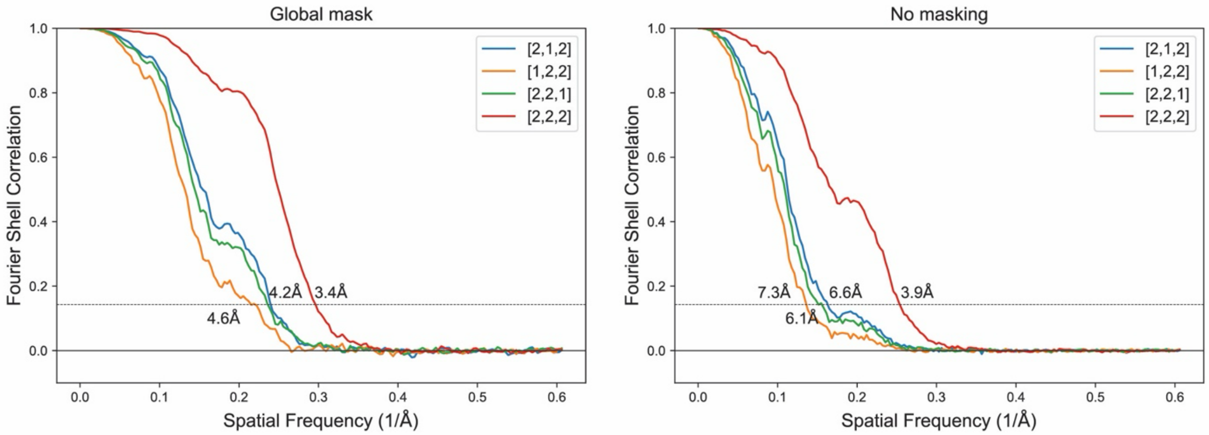

c

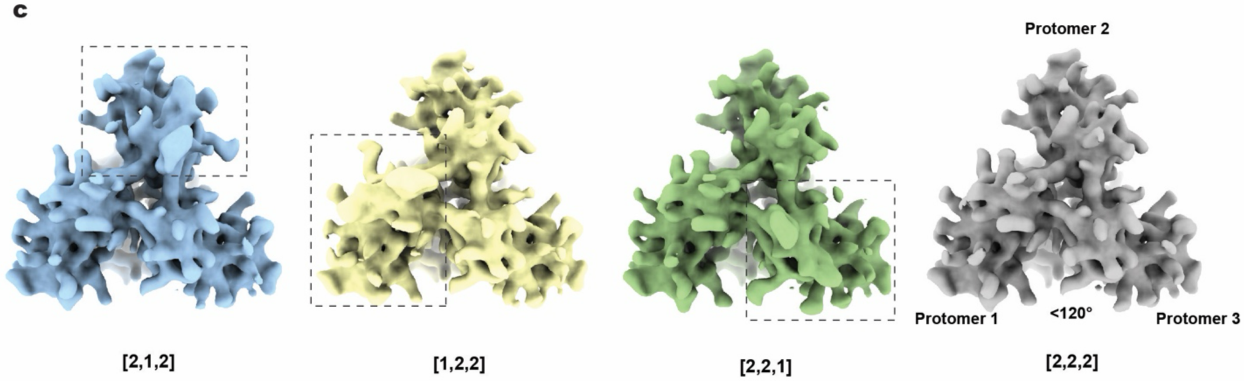

Supplementary Fig. 11. Mixed Tri FPPR Env trimers consisting of gp120.1 and gp120.2 protomers. The indicated permutations of mixed gp120.1 and gp120.2 protomers were identified in the Tri FPPR Env population. **a.** The data collection and processing statistics for the mixed gp120.1-gp120.2 Env trimers are reported. **b.** The gold-standard Fourier Shell Correlations for these trimer subclasses are shown, with resolutions estimated at an FSC cutoff of 0.143. **c.** The mixed gp120.1-gp120.2 Env maps are shown along with the map of a pure

gp120.2 Env trimer (2,2,2). The maps are viewed from the perspective of the target cell, looking at the Env trimer apex. In this orientation, the smallest opening angle is at the bottom of the image. The gp120.1 protomers in the mixed gp120.1-gp120.2 Env maps are marked with a broken rectangle.

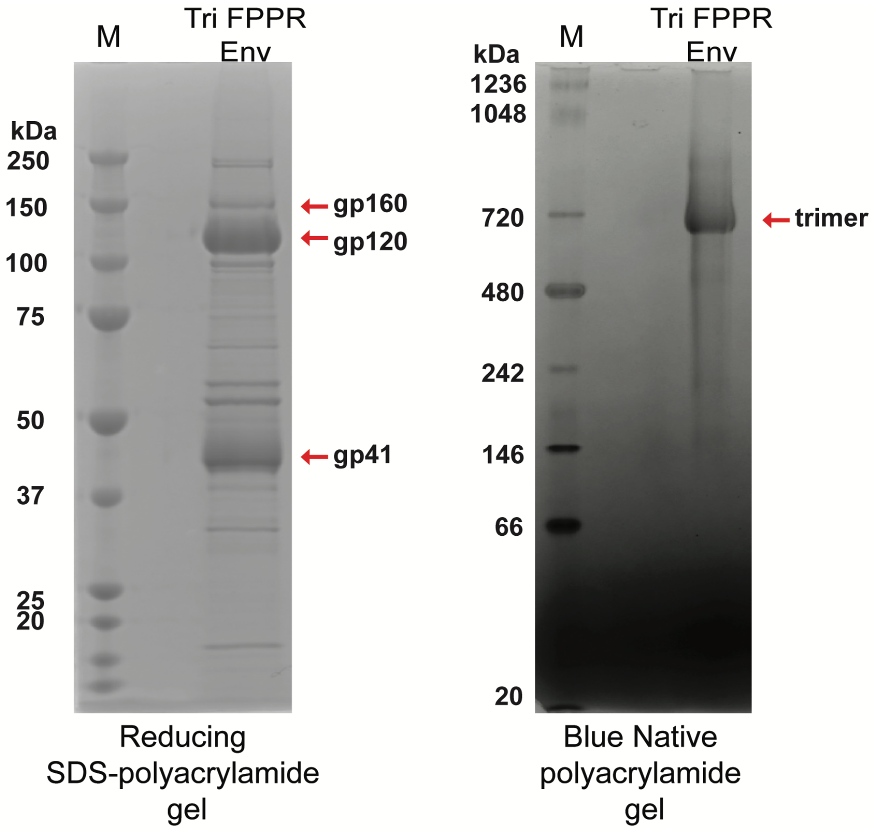

Supplementary Fig. 12. Preparation of the Tri FPPR Env trimers. The purified Tri FPPR Env was analyzed on a reducing SDS-polyacrylamide gel (left) and on a Blue Native polyacrylamide gel (right). The results are representative of those obtained in at least two independent experiments. M – molecular weight markers.

Supplementary References

1. Fu, Q. *et al.* Structure of the membrane proximal external region of HIV-1 envelope glycoprotein. *Proc Natl Acad Sci U S A* **115**, E8892-E8899 (2018).

2. Kwon, B., Lee, M., Waring, A. J. & Hong, M. Oligomeric structure and three-dimensional fold of the HIV gp41 membrane-proximal external region and transmembrane domain in phospholipid bilayers. *J Am Chem Soc* **140**, 8246-8259 (2018).

3. Yang, S. *et al.* Dynamic HIV-1 spike motion creates vulnerability for its membrane-bound tripod to antibody attack. *Nat Commun* **13**, 6393 (2022).

4. Li, Z. *et al.* Subnanometer structures of HIV-1 envelope trimers on aldrithiol-2-inactivated virus particles. *Nat Struct Mol Biol* **27**, 726-734 (2020).

5. Mangala Prasad, V. *et al.* Cryo-ET of Env on intact HIV virions reveals structural variation and positioning on the Gag lattice. *Cell* **185**, 641-653 e617 (2022).

6. Nguyen, H. T. *et al.* Functional and highly cross-linkable HIV-1 envelope glycoproteins enriched in a pretriggered conformation. *J Virol* **96**, e0166821 (2022).

7. Zhang, Z. *et al.* Membrane HIV-1 envelope glycoproteins stabilized more strongly in a pretriggered conformation than natural virus Envs. *iScience* **27**, 110141 (2024).

8. Bai, X. C., Rajendra, E., Yang, G., Shi, Y. & Scheres, S. H. Sampling the conformational space of the catalytic subunit of human gamma-secretase. *Elife* **4**, 11182 (2015).

9. Zhang, K. Gctf: Real-time CTF determination and correction. *J Struct Biol* **193**, 1-12 (2016).

10. Kucukelbir, A., Sigworth, F. J. & Tagare, H. D. Quantifying the local resolution of cryo-EM density maps. *Nat Methods* **11**, 63-65 (2014).

11. Wang, K. *et al.* Asymmetric conformations of cleaved HIV-1 envelope glycoprotein trimers in styrene-maleic acid lipid nanoparticles. *Commun Biol* **6**, 535 (2023).

12. Pancera, M. *et al.* Crystal structures of trimeric HIV envelope with entry inhibitors BMS-378806 and BMS-626529. *Nat Chem Biol* **13**, 1115-1122 (2017).

13. Korber, B.T., Foley, B.T., Kuiken, C.L., Pillai, S.K. & Sodroski, J.G. Numbering positions in HIV relative to HXB2cg. HIV Sequence Compendium 1998. Los Alamos National Laboratory. Theoretical Biology and Biophysics, Los Alamos, New Mexico.

14. Ofek, G. *et al.* Structure and mechanistic analysis of the anti-human immunodeficiency virus type 1 antibody 2F5 in complex with its gp41 epitope. *J Virol* **78**, 10724-10737 (2004).

15. Huang, J. *et al.* Broad and potent neutralization of HIV-1 by a gp41-specific human antibody. *Nature* **491**, 406-412 (2012).
16. Sun, Z.-Y. *et al*. HIV-1 broadly neutralizing antibody extracts its epitope from a kinked gp41 ectodomain region on the viral membrane. *Immunity* **28**, 52-63 (2008).
17. Haim, H. *et al*. Contribution of intrinsic reactivity of the HIV-1 envelope glycoproteins to CD4-independent infection and global inhibitor sensitivity. *PLoS Pathog* **9**, e1002101 (2011).
18. Williams, W. B. *et al.* Vaccine induction of heterologous HIV-1-neutralizing antibody B cell lineages in humans. Cell **187**, 2919-2934 (2024).
19. Pettersen, E. F. *et al*. UCSF Chimera – a visualization system for exploratory research and analysis. *J Comput Chem* **25**, 1605-1612 (2004).
