# Supplementary material for: The membrane-proximal external region of human immunodeficiency virus (HIV-1) envelope glycoprotein trimers in A18-lipid nanodiscs
Source: Commun Biol. 2025 Mar 15;8:442. doi: 10.1038/s42003-025-07852-z (PMC11910548; doi:10.1038/s42003-025-07852-z)
Supplement: Supplementary file 3 — Description of Additional Supplementary Materials [file 42003_2025_7852_MOESM3_ESM.pdf]

Description of Additional Supplementary Files

File name: Supplementary Data 1

Description: The source data for the Fourier Shell Correlation (FSC) curves shown in Supplementary Figure 3d and Supplementary Fig. 11b.
