# Supplementary material for: The membrane-proximal external region of human immunodeficiency virus (HIV-1) envelope glycoprotein trimers in A18-lipid nanodiscs
Source: Commun Biol. 2025 Mar 15;8:442. doi: 10.1038/s42003-025-07852-z (PMC11910548; doi:10.1038/s42003-025-07852-z)
Supplement: Supplementary file 4 — Reporting summary [file 42003_2025_7852_MOESM4_ESM.pdf]

nature portfolio

Corresponding author(s): Joseph Sodroski, Youdong Mao

Last updated by author(s): Feb 3, 2025

Reporting Summary

Nature Portfolio wishes to improve the reproducibility of the work that we publish. This form provides structure for consistency and transparency in reporting. For further information on Nature Portfolio policies, see our Editorial Policies and the Editorial Policy Checklist.

Statistics

For all statistical analyses, confirm that the following items are present in the figure legend, table legend, main text, or Methods section.

n/a	Confirmed	
☐	☒	The exact sample size (*n*) for each experimental group/condition, given as a discrete number and unit of measurement
☒	☐	A statement on whether measurements were taken from distinct samples or whether the same sample was measured repeatedly
☐	☒	The statistical test(s) used AND whether they are one- or two-sided<br>*Only common tests should be described solely by name; describe more complex techniques in the Methods section.*
☒	☐	A description of all covariates tested
☒	☐	A description of any assumptions or corrections, such as tests of normality and adjustment for multiple comparisons
☐	☒	A full description of the statistical parameters including central tendency (e.g. means) or other basic estimates (e.g. regression coefficient) AND variation (e.g. standard deviation) or associated estimates of uncertainty (e.g. confidence intervals)
☐	☒	For null hypothesis testing, the test statistic (e.g. F, t, r) with confidence intervals, effect sizes, degrees of freedom and P value noted<br>*Give P values as exact values whenever suitable.*
☒	☐	For Bayesian analysis, information on the choice of priors and Markov chain Monte Carlo settings
☒	☐	For hierarchical and complex designs, identification of the appropriate level for tests and full reporting of outcomes
☐	☒	Estimates of effect sizes (e.g. Cohen's *d*, Pearson's *r*), indicating how they were calculated

Our web collection on statistics for biologists contains articles on many of the points above.

Software and code

Policy information about availability of computer code

Data collection	SerialEM v3.6.11
Data analysis	MotionCor2 v1.6.3, GCTF v1.06, DeepEM v1.0, EMAN v2.91, ROME v1.1.2., RELION v3.0., Coot v0.9.8., Phenix v1.18.2, ResMap v1.19.2, Pymol v2.5.4, UCSF Chimera v1.16, ChimeraX v1.7.1

For manuscripts utilizing custom algorithms or software that are central to the research but not yet described in published literature, software must be made available to editors and reviewers. We strongly encourage code deposition in a community repository (e.g. GitHub). See the Nature Portfolio guidelines for submitting code & software for further information.

Data

Policy information about availability of data

All manuscripts must include a data availability statement. This statement should provide the following information, where applicable:

- Accession codes, unique identifiers, or web links for publicly available datasets
- A description of any restrictions on data availability
- For clinical datasets or third party data, please ensure that the statement adheres to our policy

Data supporting the findings of this manuscript are available from the corresponding authors upon reasonable request. The cryo-EM density maps have been deposited in the Electron Microscopy Data Bank (EMDB) under accession codes EMD-61553 (Tri FPPR.1) and EMD-61554 (Tri FPPR.2). The coordinates have been deposited in the RCSB Protein Data Bank (PDB) under accession codes 9JKF (Tri FPPR.1) and 9JKG (Tri FPPR.2).

Research involving human participants, their data, or biological material

Policy information about studies with human participants or human data. See also policy information about sex, gender (identity/presentation), and sexual orientation and race, ethnicity and racism.

Reporting on sex and gender	N/A
Reporting on race, ethnicity, or other socially relevant groupings	N/A
Population characteristics	N/A
Recruitment	N/A
Ethics oversight	N/A

Note that full information on the approval of the study protocol must also be provided in the manuscript.

Field-specific reporting

Please select the one below that is the best fit for your research. If you are not sure, read the appropriate sections before making your selection.

☒ Life sciences ☐ Behavioural & social sciences ☐ Ecological, evolutionary & environmental sciences

For a reference copy of the document with all sections, see nature.com/documents/nr-reporting-summary-flat.pdf

Life sciences study design

All studies must disclose on these points even when the disclosure is negative.

Sample size	No statistical methods were used to estimate appropriate sample size.
Data exclusions	No data were excluded from the analysis. During cryo-EM data clustering, good cryo-EM images were chosen for further 3D analysis based on their achieved resolution and reconstruction quality. Poorer images were excluded from the final reconstructions based on the criteria of maximizing the map resolution and quality.
Replication	Purification of the Tri FPPR Env was performed successfully more than two times.
Randomization	Single-particle datasets of cryo-EM images were randomly split for the purposes of estimating overall resolution during Fourier shell correlation calculation. Otherwise, randomization was not relevant to these studies.
Blinding	Blinding was not relevant to this study.

Reporting for specific materials, systems and methods

We require information from authors about some types of materials, experimental systems and methods used in many studies. Here, indicate whether each material, system or method listed is relevant to your study. If you are not sure if a list item applies to your research, read the appropriate section before selecting a response.

Materials & experimental systems

n/a	Involved in the study	
☐	☒	Antibodies
☐	☒	Eukaryotic cell lines
☒	☐	Palaeontology and archaeology
☒	☐	Animals and other organisms
☒	☐	Clinical data
☒	☐	Dual use research of concern
☒	☐	Plants

Methods

n/a	Involved in the study	
☒	☐	ChIP-seq
☒	☐	Flow cytometry
☒	☐	MRI-based neuroimaging

Antibodies

Antibodies used	The poorly neutralizing antibodies used in this study include 19b (gp120 V3) and F240 (gp41 Cluster I), which were obtained from

Antibodies used	James Robinson (Tulane University) and Marshall Posner (Mount Sinai Medical Center), respectively
Validation	The 19b and F240 antibodies were produced by transfection with plasmids expressing the antibody heavy and light chains; the plasmids were sequenced to confirm the identity of the antibodies.

Eukaryotic cell lines

Policy information about cell lines and Sex and Gender in Research

Cell line source(s)	Human A549 lung epithelial cells (ATCC) inducibly expressing the Tri FPPR Envs were established in the Kappes laboratory as described in Methods. See Zhang, S. et al., J Virol 95, e0052921 (2021) and Wang, K. et al., Commun Biol 6, 535 (2023).
Authentication	The sequence of the Tri FPPR env gene was confirmed.
Mycoplasma contamination	Mycoplasma was not detected in the A549 cell line.
Commonly misidentified lines (See ICLAC register)	No commonly misidentified cell lines were used in this study.

Plants

Seed stocks	No seed stocks or other plant material were involved.
Novel plant genotypes	No novel plants were involved.
Authentication	No authentication procedures for seed stocks or novel genotype were needed.
